# Linoleic Acid-Enriched Diet Increases Mitochondrial Tetralinoleoyl Cardiolipin, OXPHOS Protein Levels, and Uncoupling in Interscapular Brown Adipose Tissue during Diet-Induced Weight Gain

**DOI:** 10.3390/biology12010009

**Published:** 2022-12-21

**Authors:** Deena B. Snoke, Connor A. Mahler, Austin Angelotti, Rachel M. Cole, Genevieve C. Sparagna, Kedryn K. Baskin, Martha A. Belury

**Affiliations:** 1 Department of Medicine, Larner College of Medicine, University of Vermont, Burlington, VT 05405, USA; 2 Department of Human Sciences, College of Education and Human Ecology, The Ohio State University, Columbus, OH 43210, USA; 3 Indiana Biosciences Research Institute, Indianapolis, IN 46062, USA; 4 Department of Medicine, Division of Cardiology, University of Colorado Anschutz Medical Campus, Aurora, CO 80045, USA; 5 Department of Physiology and Cell Biology, The Ohio State University College of Medicine, Columbus, OH 43210, USA

**Keywords:** cardiolipin, brown adipose tissue, high fat diet, dietary fat, linoleic acid, metabolic syndrome, obesity, insulin sensitivity

## Abstract

**Simple Summary:**

We explored the ability of dietary fat quality to influence mitochondrial cardiolipin species, respiratory chain protein levels, and function in brown adipose tissue during the consumption of moderate-fat diets in mice. Findings from this study suggest that diets containing linoleic acid (LA)-rich oil may confer whole-body and brown adipose tissue (BAT)-specific metabolic benefits through its ability to enrich linoleic acid-containing cardiolipin (CL) species to best support mitochondrial uncoupling.

**Abstract:**

Cardiolipin (CL) is a phospholipid unique to the inner mitochondrial membrane that supports respiratory chain structure and function and is demonstrated to be influenced by types of dietary fats. However, the influence of dietary fat on CL species and how this best supports mitochondrial function in brown adipose tissue (BAT), which exhibits an alternative method of energy utilization through the uncoupling of the mitochondrial proton gradient to generate heat, is not well understood. Therefore, the aim of our study was to evaluate metabolic parameters, interscapular BAT CL quantity, species, and mitochondrial function in mice consuming isocaloric moderate-fat diets with either lard (LD; similar fatty acid profile to western dietary patterns) or safflower oil high in linoleic acid (SO), shown to be metabolically favorable in large clinical meta-analyses. Mice fed the SO diet exhibited decreased adiposity, improved insulin sensitivity, and enrichment of LA-containing CL species in BAT CL. Furthermore, mice fed the SO diet exhibit higher levels of OXPHOS complex proteins and increased oxygen consumption in BAT. Our findings demonstrate that dietary consumption of LA-rich oil improves metabolic parameters, increases LA-containing CL species, and improves BAT function when compared to the consumption of lard in mice during diet-induced weight gain.

## 1. Introduction

Cardiolipin (CL) is a glycerophospholipid unique to mitochondrial and bacterial membranes [1]. Its unique structure, comprised of two glycerol backbones, two phosphatidic acid head groups, and four acyl chains confers a negative charge, allowing for it to provide structural support for the formation of cristae and the alignment of oxidative phosphorylation (OXPHOS) complexes required for ATP production in the inner mitochondrial membrane (IMM) [2,3,4]. CL is well studied for its role in the mitochondrial dynamics of the heart, where 80–90% of acyl chains are the essential omega-6 fatty acid (FA), linoleic acid (18:2n6; LA), and roughly 80% of CL contains 4 LA (tetralinoeoyl; L_4_CL) [5,6,7]. Changes to CL speciation and regulation of its remodeling have been implicated in a number of metabolic conditions associated with metabolic syndrome and obesity, including heart failure, diabetic cardiomyopathy, and malignancies [4]. Although efficient mitochondrial function is an essential process driving many cellular events that could alter whole-body energy metabolism, information about the role of CL speciation and how it contributes to this process in many tissues is limited.

Recent discoveries highlight the role of brown adipose tissue (BAT) as a powerful endocrine organ regulating whole-body metabolic homeostasis [8,9]. Contrary to the classical function of white adipose tissue as an energy storage reservoir, BAT produces heat, rather than ATP, through the activity of Ucp-1 which eliminates the proton gradient generated from OXPHOS complexes in the IMM. It is no surprise that similar to other IMM proteins requiring structural stabilization, Ucp-1 binds to three CL molecules [10,11]. CL production is also driven by the upregulation of thermogenesis during cold exposure and increased CL is a biomarker of thermogenic adipose tissues [12]. Additionally, CL in BAT was demonstrated to be necessary for whole-body insulin sensitivity, as its loss leads to insulin resistance [13]. Together, these findings highlight the indispensable role of CL, not only in BAT mitochondrial function, but also in its contribution to whole-body energy metabolism. 

Dietary fat quality may affect CL quantity and species [14,15], with a majority of studies focusing on mitochondrial CL in the liver and heart [14]. Despite several recent reports of the beneficial metabolic effects of dietary LA consumption in large patient populations [16,17,18,19,20], few studies have explored how dietary FA composition affects mitochondrial function in other mitochondrial-rich tissues, such as BAT, and how this might contribute to insulin sensitivity.

Due to its physiological role in affecting whole-body energy metabolism, BAT possesses the great therapeutic potential to alleviate the health and economic burdens of a constellation of metabolic conditions associated with obesity [8,9,21,22]. Therefore, we explored the effect of dietary fat quality on CL speciation and mitochondrial function in BAT to test the hypothesis that a diet higher in LA would confer favorable changes to CL speciation and improve BAT mitochondrial function compared to a lipid profile more similar to current western dietary patterns. To address this question, wild-type mice were fed moderate-fat diets (24% by weight, 45% kcal) differing only in their fat source (lard or high-linoleic safflower oil) and thus lipid composition for a period of 14–18 weeks to achieve diet-induced weight gain. BAT CL species and measures of mitochondrial health and function were measured and the effects of these diets on physiological measurements of metabolic health, as well as dietary fat-induced changes to CL speciation and mitochondrial function in BAT are reported. 

## 2. Materials and Methods

### 2.1. Mouse Model and Experimental Diet Composition

Twenty-four 9-week- old male C57BL/J6 mice (N = 24) were obtained from Jackson Laboratory (Bar Harbor, MA, USA). Upon arrival, the animals were moved into individual housing in a vivarium with a room temperature of 22 ± 0.5 °C, a 12-h light/dark cycle, and free access to food and water. Mice were acclimated for two weeks in cages equipped with enrichment objects and consumed a chow diet. Post-acclimation, mice were randomized into two diet groups: lard (LD) or safflower oil (SO; n = 12/group). Each diet was a semi-purified moderate fat diet, consisting of 24%w/w protein, 41%w/w carbohydrate, and 24% w/w fat (45% kcal; Supplementary Table S1), where the fat source of the LD diet was lard (D20012819, Research Diets Inc. New Brunswick, NJ, USA) and that of the SO safflower oil diet was high-linoleic safflower oil (D19081203B, Research Diets Inc., New Brunswick, NJ, USA; Supplementary Table S1). Body weight and food intake were measured every other day. Mice consumed diets for a total of 14–18 weeks. 

**Figure 1:**
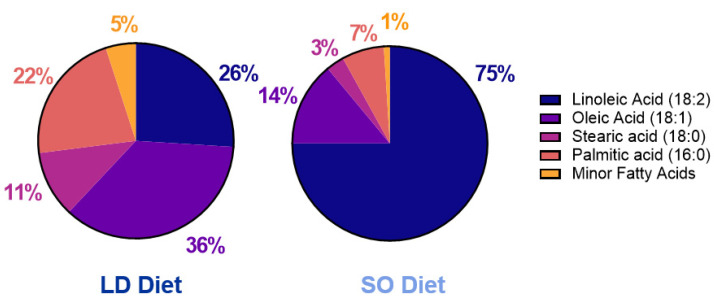
Fatty acid composition of the fat sources used for LD and SO diets. Dietary oils (n = 3 samples/group) were analyzed by gas chromatography and expressed as a percent of total fatty acids. All samples have a coefficient of variation of <3% between experimental replicates.

Total lipids were extracted from diet samples with 2:1 (*v*/*v*) chloroform: methanol [23]. FA methyl esters were prepared using 5% hydrochloric acid in methanol [24]. Analysis of FA methyl esters was completed by gas chromatography as previously described [25] and reported as a percent of the total identified. FA composition of LD and SO diet fat sources (LA-rich safflower oil, and lard, respectively) are found in Figure 1 and reported in Supplementary Table S2. 

Mice were euthanized under isoflurane anesthesia with cervical dislocation between study days 101–121. Those mice used for the mitochondrial function were anesthetized beginning on Day 101. Adipose depots, skeletal muscles, and heart were weighed, flash frozen in liquid nitrogen, and stored at −80 °C until further analysis. Half of the interscapular BAT depot was immediately flash frozen in liquid nitrogen, while the other half was collected and processed for mitochondrial functional assays as described below. All procedures were approved by The Ohio State University Institutional Animal Care and Use Committee (protocol # 2013A00000036-R2) and have therefore been performed in accordance with the ethical standards laid down in the 1964 Declaration of Helsinki and its later amendments.

### 2.2. EchoMRI for Body Composition

To assess time-course changes in the body composition of live mice, EchoMRI (Houston, TX, USA) was used to measure lean body weight and adiposity at three timepoints: Day 0, Day 65, and Day 100. Percent adiposity and percent lean body mass were calculated relative to each animal’s body weight on the day of the procedure.

### 2.3. Grip Strength

Mice were acclimated to forelimb and hindlimb grip strength testing 3 consecutive times during the week prior to the first experimental measurement. Forelimb and hindlimb grip strength were measured on Day 113 with the Columbus Instruments Grip Strength Meter (Columbus, OH, USA). All practice and experimental measurements on the grip strength meter were taken in triplicate, with at least one minute of rest between each measurement.

### 2.4. Insulin-Stimulated Glucose Uptake 

An insulin tolerance test (ITT) was used to assess insulin sensitivity on Day 85. Mice were moved to clean cages without food and bedding to a quiet room for 6 h prior to the ITT. Briefly, mice were pricked at the tip of the tail with a 20-gauge needle and blood glucose was measured using the OneTouch Ultra glucometer (Lifescan, Inc., Milpitas, CA, USA). After measuring blood glucose, mice were given a bolus of 0.75 U/kg insulin by intraperitoneal injection. Blood glucose was measured at 0, 15, 30, 45, 60, 90, and 120-min post-insulin injection. 

### 2.5. Mitochondrial Isolation

Mitochondria were isolated as described previously [26,27,28,29,30,31]. Briefly, interscapular BAT was harvested, minced, and placed in an ice cold MSHE buffer (70 mM sucrose, 210 mM mannitol, 1 mM HEPES, 1 mM EGTA, 0.5% FA-free bovine serum albumin (BSA, MP Biomedicals); pH was adjusted to 7.2 using potassium hydroxide). Minced tissue was homogenized at 4 °C using an electronic tissue grinder and centrifuged at 800× *g* at 4 °C for 10 min. The supernatant was filtered through a multilayered cheesecloth and centrifuged at 8000× *g* at 4 °C for 10 min. The resulting pellet was resuspended in MSHE buffer and centrifuged at 8000× *g* at 4 °C for 10 min. This pellet was resuspended in a small volume of MSHE without BSA, centrifuged at 8000× *g* at 4 °C for 5 min, and the final pellet was resuspended in a minimal volume of MSHE without BSA (30–50 µL). 

### 2.6. Cardiolipin Speciation 

CL was quantified from 25–50 mg protein BAT mitochondria per sample using previously published methods with normal phase liquid chromatography coupled to electrospray ionization mass spectrometry in an API 4000 mass spectrometer (Sciex, Framingham, MA, USA) [32]. Lipids were extracted by the modified Bligh Dyer method according to previously published methods with 1000 nmol tetramyristal CL as an internal standard (Avanti Polar Lipids, Alabaster, AL, USA) [33].

### 2.7. Gene Expression (qPCR)

Approximately 50–75 mg of BAT were cut on dry ice. BAT mRNA was isolated (RNeasy Lipid Tissue Mini Kit, Qiagen, Hilden, Germany) according to the manufacturer’s protocol and included the optional DNase digest. RNA concentration was measured (NanoDrop1000, Thermo Scientific, Waltham MA, USA); RNA with a concentration greater than 200 ng and 260/230 spectra values of greater than 1.8 were used for reverse transcription and subsequent real-time quantitative PCR analysis. RNA was reverse transcribed to cDNA (High-Capacity cDNA Archive Kit, Applied Biosystems, Foster City, CA, USA). cDNA was amplified by qPCR with TaqMan Gene Expression Assays using predesigned and validated primers under universal cycling conditions defined by Applied Biosystems. Target gene expression was normalized to an endogenous control cyclophilin A (mouse PPIA; Mm02342430_g1); peroxisome proliferator-activated receptor γ (PPAR γ; Mm00440940_m1) well and cell death-inducing DNA fragmentation factor, alpha subunit-like (*Cidea*; Mm00423554_m1), peroxisome proliferator-activated receptor coactivator- 1α (*PGC-1α*; Mm01208835_m1), lipoprotein lipase (*Lpl*; Mm00434764_m1), uncoupling protein 1 (*Ucp1*; Mm01244861_m1), Cardiolipin Synthase 1 (*Crls1*; Mm01278100_m1), and taffazin (*Taz*; Mm00504978). All target genes were amplified in the same reaction wells as the endogenous control genes, and are expressed as 2^−ΔΔCT^ relative to the control group [34]. 

### 2.8. OXPHOS Protein Complex Immunoblotting 

Protein concentrations of isolated mitochondria samples were determined by BCA Protein Assay Kit according to the vendor protocol (Pierce Chemical Co., Dallas, TX, USA). For total OXPHOS protein detection, 5 μg of protein from each isolated mitochondria sample was separated using sodium dodecyl sulfate polyacrylamide gel electrophoresis with a 15% SDS-PAGE gel. Protein was transferred to a PVDF membrane in transfer buffer (25 mM Tris, 192 mM glycine, 20% methanol; Bio-Rad Laboratories, Hercules, CA, USA). After drying the membrane for 1 h at room temperature, membranes were incubated in Revert 700 total protein stain (LI-COR Biosciences, Lincoln, NE, USA) and total protein was detected as a loading control using the LI-COR Odyssey Imager (LI-COR Biosciences, Lincoln, NE, USA). The membrane was then blocked with 5% nonfat dry milk in Tris-Buffered Saline for 1 h and incubated on a shaker overnight at 4 °C with primary antibody (1: 500; Total OXPHOS; Abcam, Cambridge, United Kingdom). After washing, the membrane was incubated for 1 h with fluorophore-conjugated secondary antibodies and bands were detected using the LI-COR Odyssey Imager (LI-COR Biosciences, Lincoln, NE, USA). Densitometric Analysis was carried out using the Licor Imager Software (LI-COR Biosciences, Lincoln, NE, USA). 

### 2.9. Oxygen Consumption Rate and Mitochondrial Stress Test 

#### 2.9.1. Seahorse XFe96 Assay on Isolated Mitochondria

Electron flow (EF) assays were performed using the XFe96 Extracellular Flux Analyzer (Seahorse Bioscience). Mitochondria isolated from BAT were resuspended at optimal concentrations in EF buffer comprised of MAS1 buffer (70 mM sucrose, 220 mM mannitol, 5 mM KH_2_PO_4_, 5 mM MgCl_2_, and 2 mM HEPES, 1 mM EGTA, pH 7.2). Isolated mitochondria were resuspended in an EF assay buffer comprised of MAS1 buffer supplemented with 10 mM pyruvate, 2 mM malate, 4 µM FCCP, and 0.5% BSA, pH 7.2. EF buffer without BSA was used to prepare optimized concentrations of rotenone (port A), succinate (port B), antimycin A (port C), and TMPD (N, N, N′, N′-tetramethyl-p-phenylenediamine) (port D). Stock concentrations for succinate (500 mM) and ADP (100 mM) were prepared in molecular biology-grade water. Rotenone (1 mM), Oligomycin (20 mM), FCCP (10 mM), and Antimycin A (40 mM) stock concentrations were prepared in DMSO. TMPD (10 mM) was prepared in 10 mM ascorbate diluted in reagent-grade water. All reagents were adjusted to a pH of 7.2. For EF assays, three sequential measurements were taken to determine oxygen levels and pH at baseline and after injection of rotenone, succinate, antimycin A, and TMPD. 

#### 2.9.2. Mitochondrial DNA (mtDNA) Isolation

An mtDNA isolation protocol was performed as previously described [29]. After the Seahorse assays were run, the buffer was removed from the assay plate. Trizol (50 mL) was added to each well and the contents were moved to microcentrifuge tubes. Chloroform was added, the contents vigorously mixed, and centrifuged for 15 min at 12,000× *g*, 4 °C. The aqueous layer containing RNA was discarded. Back extraction buffer (4M guanidine thiocyanate, 50 mM sodium citrate, 1M Tris base) was added to the interphase and organic phase, and the samples were inverted several times. After a 10-min incubation at room temperature, samples were centrifuged for 30 min at 300× *g*, 4 °C. The upper phase containing DNA was transferred to new tubes and 1 μL polyacryl carrier was added (Molecular Research Center PC152) followed by isopropanol. Tubes were inverted several times and incubated at room temperature for 5 min. DNA was pelleted at 12,000× *g* for 5 min at 4 °C. The pellet was washed 3 times with 70% ethanol. After the final wash, pellets were air dried and resuspended in a minimal volume of 8 mM NaOH and vortexed. A mix of 1M HEPES and 100 mM EDTA was added to each isolated DNA. DNA samples were incubated at room temperature overnight and DNA concentrations measured. 

#### 2.9.3. Mitochondrial DNA (mtDNA) Quantification

A quantitative polymerase chain reaction (qPCR) was performed on representative technical replicates to validate relative amounts of mitochondria between samples. A qPCR was performed according to vendor protocol with iTaq Universal Sybr green reagent (Bio-Rad) on 5 ng of mtDNA per sample for NADH dehydrogenase subunit 1 (mtND1; forward primer 5′-CCC ATT CGC GTT ATT CTT-3′ and reverse primer 5′-AAG TTG ATC GTA ACG GAA GC-3′) and mitochondrial ribosomal RNA (mt16S; forward primer 5′-CCG CAA GGG AAA GAT GAA AGA C-3′, and reverse primer 5′-TCG TTT GGT TTC GGG GTT TC-3′). Ct values were compared between samples. 

### 2.10. Statistical Analyses 

Data were assessed for equal variance between groups for each outcome prior to representation as the mean ± the standard error of the mean (SEM). Differences between LD and SO groups were analyzed by two sample *t*-tests. Statistical outliers were calculated by comparing individual values to a cutoff range calculated as mean ± 3 (SD). All statistical tests were performed using STATA (StataCorp LLC, College Station, TX, USA) and graphed with Graphpad Prism (Graphpad Software, San Diego, CA, USA). All statistical tests were performed at the 5% significance level.

## 3. Results

### 3.1. Effect of LD and SO Diets on Physiological Parameters 

**Figure 2:**
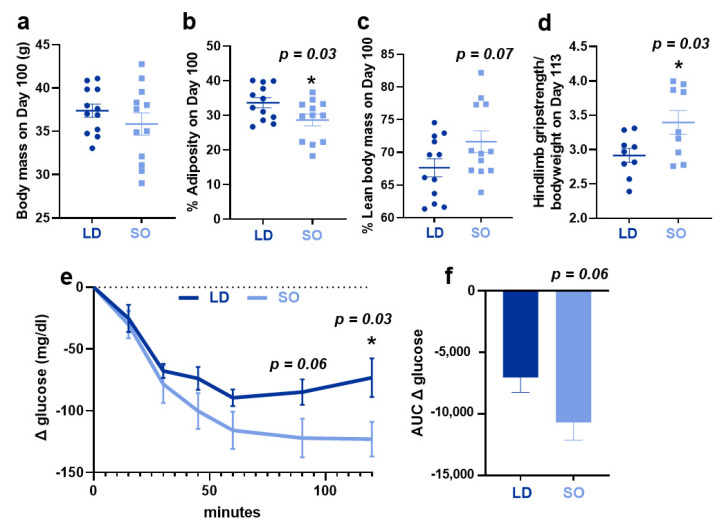
Physiological parameters of mice consuming LD and SO diets for 14–18 weeks. (**a**) Body mass on Day 100 of the study, prior to necropsy. (**b**) Percent adiposity as measured by EchoMRI on day 100. (**c**) Percent lean body mass as measured by EchoMRI on day 100. (**d**) Hindlimb grip strength as measured by Grip Strength Meter on Day 113. (**e**) On day 85, mice were given a bolus of 0.75 U/kg of insulin injected intraperitoneally, blood glucose was measured over a period of 120 min, and (**f**) the area under the curve for glucose clearance was computed. Data are expressed as individual values and the graph displays the mean +/− SEM. Two-sample *t*-tests were used to determine significant differences between diet groups. For all statistical analyses, an asterisk (*) designates significance with *p* < 0.05, while the absence of asterisks indicates that the *p*-value was nonsignificant. The *p*-values < 0.10 are listed in each figure. N = 9–12/group.

Consumption of LD (SFA:MUFA:PUFA 2:2:1) or SO (SFA:MUFA:PUFA 1:1:5; see Figure 1 and Table S1 for diet composition) diets for a period of 14–18 weeks led to weight gain in both LD and SO diet groups over time (data not shown). However, there was no difference in food intake, body weight, or tissue weights between groups at the end of the study (Figure 2a; Supplementary Table S3). Despite this, there were differences in body composition between groups: the SO diet had an effect to lower the percent of adiposity (*p* = 0.03) and a trend toward higher lean body mass (*p* = 0.07) compared to the LD diet (Figure 2b,c). Despite little change to lean body mass, mice in the SO group exhibited increased hindlimb grip strength at the end of the study compared to those in the SO group (Figure 1d; *p* = 0.03). The LD diet attenuated glucose clearance after insulin administration, particularly at later time points post-insulin injection (Figure 1e). However, this did not translate to a significantly greater area under the curve (Figure 2f; *p* = 0.06). 

### 3.2. Effect of LD and SO Diets on Total CL and CL Species and Gene Expression of CL Remodeling Enzymes in BAT

**Figure 3:**
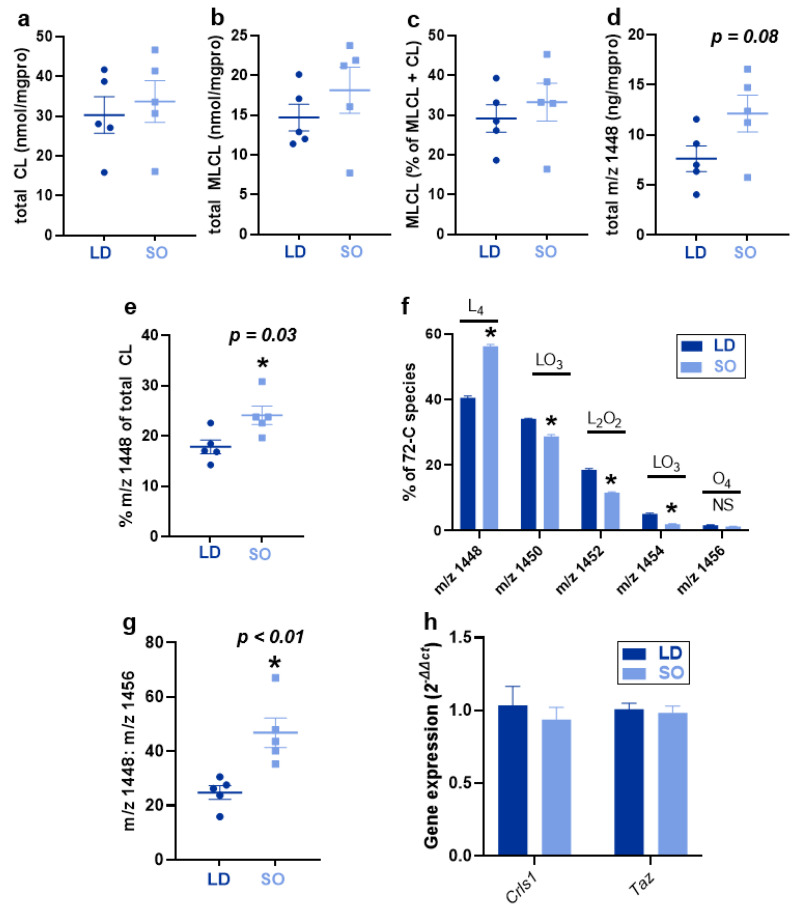
Effect of LD and SO diet consumption on total CL, monolyso-CL, 72-carbon CL species, and gene expression of CL synthesis and remodeling enzymes in BAT. CL was measured using normal phase liquid chromatography coupled with electrospray ionization mass spectrometry and reported relative to quantified protein levels in the sample. (**a**) Total CL. (**b**) Total monolyso-CL (MLCL). (**c**) MLCL as a percent of the total MLCL + CL in each tissue sample. (**d**) Total mass:charge (*m*/*z*) 1448 (predominant acyl species L_4_CL) and (**e**) Am/z of 1448 as a percent of the total CL in each tissue sample. (**f**) The 72-carbon species of CL in each diet group expressed as a percent of the total 72C species with the major species of CL for each *m*/*z* value written above each set of bars. (**g**) Ratio between *m*/*z* 1448 and *m*/*z* 1456 (predominant acyl species O_4_CL). (**h**) Gene expression of cardiolipin synthase (*Crls1)* and tafazzin (*Taz)*, expressed as the relative fold change (2^−ΔΔct^). For all data, data are expressed as the mean + SEM. A two-sample *t*-test was used to determine significant differences between diet groups. An asterisk (*) designates *p* < 0.05, with *p*-values for < 0.10 reported in the figure. N = 5/group.

Experimental diets impacted some species of CL, with the SO diet enriching the LA content of CL. However, there was no effect of diet on total CL (Figure 3a) or the total amount of monolyso-CL (MLCL; Figure 3b), a CL species containing three fatty acyl groups formed during nascent CL synthesis, remodeling, and degradation [35]. Similarly, there was no effect of diet on L_3_MLCL, expressed as a percentage of total MLCL and CL species (Figure 3c). L_4_CL, the predominant CL species in the heart, exhibited a trend towards increasing in BAT of mice fed the SO diet compared to those fed the LD diet (Figure 3d), and was increased as a percent of total CL (Figure 3e). There was a shift in 72-carbon containing species comprised of four 18-carbon chains of oleate or LA, where mice consuming the SO diet exhibited greater relative quantities of LA containing species when expressed as a percent of the total 72-carbon species (Figure 3f), and an increased relative amount of L_4_CL compared to O_4_CL (Figure 3g). 

In addition to measuring total CL and LA-rich CL, the most abundant CL species in the BAT of mice fed the LD and SO diets were measured. There was an overall enrichment in the LA-containing CL species in mice consuming the SO diet compared to the CL species in mice consuming the LD diet (Table 1). In contrast, consumption of the LD diet led to a higher abundance of CL species in BAT containing 16-carbon FAs, palmitoleate (16:1), and palmitate (16:0). This was in stark contrast to the preferences toward longer-chain FAs secondary to the incorporation of 18-carbon ones in the top CL species in other tissues, including heart and skeletal muscle mitochondria (Supplementary Tables S4 and S5), as well as in white adipose tissues (Supplementary Tables S6 and S7), which all exhibit a preference toward 18–20-carbon chains over the incorporation of 16-carbon chains. 

### 3.3. Effect of LD and SO Diets on Mitochondrial Function in BAT

**Figure 4:**
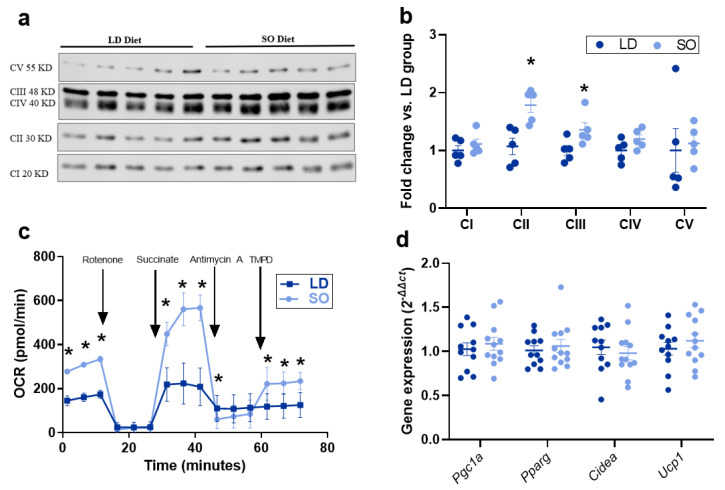
Effect of LD and SO diets on mitochondrial function in BAT. (**a**) Western blot of respiratory complexes from mitochondrial BAT extracts; CI: Complex I, NADH-coenzyme Q oxidoreductase; CII: Complex II, Succinate-Q oxidoreductase; CIII: Complex III, Q-cytochrome-C oxidoreductase; CIV: Complex IV, Cytochrome C oxidase; CV: Complex V; ATP synthase (N = 5/group). (**b**) Quantification of average respiratory complex fold change for each protein abundance, standardized to total protein as a loading control, found in Figure S1, in the SO diet compared to the LD diet (N = 5/group). (**c**) Electron flow assay to measure uncoupled respiration in BAT mitochondrial extracts expressed as an average for individual timepoints over a 72-min period. (**d**) Gene expression for *Pgc1a*, *Pparg*, *Cidea*, and *Ucp1*, expressed as relative to the LD group (N = 11–12/group). (**b**,**d**) Data expressed as the average ± SEM (-c) data expressed as the average of two independent experiments with two mitochondria samples each, ± SD. Differences between LD and SO diet groups were assessed by a two-sample *t*-test, where an asterisk (*) indicates *p* < 0.05.

To assess the effect of diet on mitochondrial activity in BAT, protein levels of the respiratory chain (OXPHOS) complexes in isolated mitochondrial extracts were measured and standardized to total protein (Figure S1). Protein levels of OXPHOS Complex II (Succinate-Q oxidoreductase) and Complex III (Q-cytochrome-C oxidoreductase) were significantly increased in the SO diet group (Figure 4a,b). To determine the influence of LD and SO diets on mitochondrial function in BAT, an electron flow assay was carried out on freshly isolated mitochondria. Complex II-dependent respiration was more than two-fold higher in the SO diet compared to the LD diet, and Complex IV respiration was slightly elevated (Figure 4c). Despite the effect of the SO diet to exhibit slightly lower Complex III-independent respiration, this still resulted in a nearly two-fold higher net basal respiration compared to the LD diet (Figure 4c). To confirm these findings, it was determined that there were no significant differences between mitochondrial DNA quantity between LD and SO diet groups, demonstrating that the increases seen in respiration in the SO diet were not due to increased mitochondrial number (Figure S2). To determine whether these differences may be due to the transcriptional control of mitochondrial biogenesis or increased markers of uncoupling, gene expression of *Pgc1a*, *Pparg*, *Cidea*, and *Ucp1* from whole BAT tissue was measured, and no effect of diet on gene expression of these markers was identified (Figure 4e). 

## 4. Discussion

The intent of this study was to identify the effect of dietary fat quality on BAT CL speciation and mitochondrial activity, and insulin-mediated glucose clearance parameters during the growth of mice. To address this goal, wild-type mice were fed moderate fat (45% kcal) LD and SO diets, differing only in their fat source and FA composition (Figure 1) for a period of 14–18 weeks. Afterward, metrics of whole-body metabolic health and assessment of CL speciation and mitochondrial activity in BAT were carried out. To our knowledge, this is the first study investigating the influence of dietary fat on CL speciation and metabolic function in BAT. 

Although there was no difference between the effect of the LD and SO diets on body mass, SO diet consumption resulted in lower percent adiposity and a trend toward increased lean body mass compared to LD diet consumption. While both groups of mice were responsive to insulin, SO mice exhibited an extended period of low blood glucose levels after insulin injection compared to LD mice. Although the current study was not designed to investigate the independent effect of BAT on insulin sensitivity, studies in preclinical models have demonstrated that BAT-specific genetic ablation of CL synthesis is sufficient to cause whole-body insulin resistance [13] and that BAT-derived lipid mediators have endocrine functions to improve insulin sensitivity and endocrine function in the muscle and the heart [36,37]. Interestingly, CL has been demonstrated to be a precursor to noncanonically formed mitochondrial oxylipins through the activity of iPLA2γ [38] and the LOX activity of Cytochrome C [39], including 13-KODE, shown to be inversely correlated to HOMA-IR [40]. Thus, future studies could define relationships between CL species and oxylipins that may originate from mitochondrial CL, and the influence of dietary fat on these outcomes. 

Chow diets can exhibit tremendous variation in overall composition and in the FAs present between batches [41]. These inconsistencies make it difficult to tease apart individual effects of diet and physiological function, especially when studying those classes of lipids heavily influenced by dietary fats. To this end, we recently showed that dietary fat quality provided in semi-purified diets can have a profound impact on oxylipin metabolites with known physiological functions in both white adipose tissue and BAT after only five weeks of consumption [42]. With the knowledge that dietary FA composition has been shown to alter CL quantity and species [14,15], it was no surprise that in the current study, dietary FA quality had an enormous impact on CL speciation in BAT. Notably, while total CL in BAT was unaltered by diet, L_4_CL was increased by 10% of the total most abundant CL species in the SO diet group compared to the LD diet group. Additionally, there was a decrease in oleate (18:1) and palmitoleate (16:1) containing species, likely explained by the decrease in oleic and palmitic acids in the diet, respectively, at the expense of increased LA content. 

Independent of diet, BAT CL exhibited a preference toward 16-carbon FA chains over longer-chain FAs, which are incorporated prior to 16-carbon FAs in cardiac muscle, skeletal muscle, and white adipose tissues. A possible explanation is that UCP-1 proton transport is dependent upon the presence of 20-carbon free FAs [43]. Thus, CLs in BAT might contain more 16-and 18-carbon chains because 20-carbon FAs are less available due to their use for such processes essential to uncoupling, thereby increasing mitochondrial activity. It is important to note that this process could be regulated, or it could be based on FAs available for incorporation and is a topic of interest for future studies. 

Finally, the effect of LD and SO diets on respiratory chain protein levels and uncoupling function was investigated. Because CL associates closely with OXPHOS proteins, we investigated the effect of these diets on OXPHOS protein levels and identified significant increases in protein levels of OXPHOS complexes II and III, with a striking nearly 2-fold increase in Complex II in the SO group compared to the LD group. These data were complemented by our functional data showing that basal respiration and Complex II-dependent respiration were increased in mitochondria from BAT in the SO group compared to that from the LD group and that these changes were independent of transcriptional regulation of cardiolipin remodeling, mitochondrial biogenesis, or adipocyte expansion. Due to the possibility that CL speciation may impact mitochondrial membrane structure, this warrants the design of future studies to assess metrics of mitochondrial structure or study the regulatory control of OXPHOS protein levels. Interestingly, while CL associates with Ucp-1 and Complexes I and III-V, it does not associate with Complex II, however, it is still necessary for super complex formation, as it aids in the folding of the membrane to facilitate the interaction of respiratory complexes I-IV, and reduces reactive oxygen species generated from the activity of Complex II [44,45,46,47]. It is indeed possible that while L_4_CL is biologically favorable, LA availability may also increase the relative quantity of LA-derived FAs produced through its downstream metabolism to generate long-chain FAs that are available to support UCP-1 function, thereby increasing uncoupled respiration. While the current study was not designed to address this question, this topic is of great interest to the scientific community for its therapeutic potential and warrants future consideration. 

## 5. Conclusions

In the current study, we explored the effect of dietary fat quality on BAT mitochondrial CL and uncoupling activity in BAT by using two moderate-fat diets differing only in their fat source: lard (LD), which is more similar to the fats present in western dietary patterns, or high-linoleic safflower oil (SO), rich in polyunsaturated LA. We identified that mice consuming the SO diet for a period of 14+ weeks exhibited decreased adiposity and prolonged insulin-induced suppression of blood glucose compared to the LD diet, demonstrating a whole-body metabolic advantage to consuming higher quantities of LA during diet-induced weight gain. Consumption of the SO diet had a dramatic effect on the enrichment of LA-containing CL species, the increase in levels of OXPHOS complex proteins, and the increase in uncoupled mitochondrial respiration in BAT compared to mice fed the LD diet. These findings suggest that the dietary enrichment of LA results in increased LA-rich CL species, which may in turn provide optimal support of respiratory complexes and uncoupling machinery to support more efficient mitochondrial function in BAT. Findings from this study also suggest the careful consideration of controlling for diet in studies pertaining to lipids and endocrine metabolism. This work underscores that improving dietary fat quality may be an effective and accessible therapeutic treatment to enhance BAT function and improve metabolic disease risk, and warrants the design of future studies to identify dietary fat-modulated structure-function relationships between CL species and improved mitochondrial activity in BAT. 

## Figures and Tables

**Table 1:** Predominant CL species in murine brown adipose tissue mitochondria after 14–18-week consumption of LD and SO diets.

CL *m*/*z* Ratio	Predominant Acyl Species	LD Diet (% of Total CL)	SO Diet (% of Total CL)	*p*-Value
1448	(18:2)_4_	25.1 ± 0.78	36.1 ± 0.28 *	**<0.01**
1450	(18:1) (18:2)_3_	21.1 ± 0.51	18.5 ± 0.53 *	**<0.01**
1452	(18:1)_2_ (18:2)_2_	11.5 ± 0.32	7.45 ± 0.15 *	**<0.01**
1424	(16:1) (18:1) (18:2)	5.65 ± 0.45	8.20 ± 0.37 *	**<0.01**
1426	(16:1) (18:1)_2_ (18:2)	6.92 ± 0.48	6.57 ± 0.18	0.52
1428	(16:1) (18:1)_3_	4.86 ± 0.27	2.75 ± 0.14 *	**<0.01**
1422	(16:1) (18:2)_3_	4.00 ± 0.54	3.40 ± 0.18	0.32
1400	(16:1)_2_ (18:1)_2_	2.32 ± 0.27	3.26 ± 0.23 *	**0.03**
1402	(16:0) (16:1) (18:1)_2_	2.65 ± 0.27	1.80 ± 044 *	**0.02**
1454	(18:1)_3_ (18:2)	3.12 ± 0.15	1.30 ± 0.11 *	**<0.01**

Data are presented as an average percent of each CL species of total CL, with each species listed as itsmass to charge (*m*/*z*) ratio andthe predominant acyl species, ± SEM; a two-sample *t*-test was used to determine significant differences (*p* < 0.05) in CL species between diet groups. Asterisks (*) indicate a significant change in CL species between diet groups with *p* < 0.05; respective *p*-values are listed. N = 5 per group. Abbreviations: 18:2, linoleate; 18:1, oleate, 16:1, palmitoleate; 16:0, palmitate.

## Data Availability

Data is contained within the article and supplementary material.

## Supplementary Materials

The following supporting information can be downloaded at: https://www.mdpi.com/article/10.3390/biology12010009/s1, Table S1: Diet composition of Lard (LD) and Safflower Oil (SO) diets; Table S2: Fatty acid profile of LD and SO diets; Table S3: Tissue weight expressed as a percentage of total body weight and food consumption characteristics of mice after long-term feeding of LD and SO diets; Table S4: Predominant CL species in murine heart mitochondria during long-term consumption of LD or SO diets; Table S5: Predominant CL species in murine gastrocnemius plantaris mitochondria during long-term consumption of LD or SO diets; Table S6: Predominant CL species in murine inguinal white adipose tissue (iWAT) during long-term consumption of LD or SO diets; Table S7: Predominant CL species in murine epididymal white adipose tissue (eWAT) during long-term consumption of LD or SO diets; Figure S1: Total protein stain used as a loading control for OXPHOS western blot on BAT mitochondrial extracts; Figure S2: Mitochondrial DNA quantification of *mtNd1* and *mt16S* from representative technical replicates to validate relative amounts of mitochondria between samples.
