# Supplementary material for: Linoleic Acid-Enriched Diet Increases Mitochondrial Tetralinoleoyl Cardiolipin, OXPHOS Protein Levels, and Uncoupling in Interscapular Brown Adipose Tissue during Diet-Induced Weight Gain
Source: Biology (Basel). 2022 Dec 21;12(1):9. doi: 10.3390/biology12010009 (PMC9854875; doi:10.3390/biology12010009)
Supplement: Supplementary file 1 [file biology-12-00009-s001.zip › biology-2036622-supplementary.pdf]

Supplementary Materials

Table S1. Diet composition of Lard (LD) and Safflower Oil (SO) diets.

Ingredient, % (grams)	Lard (LD) diet	Safflower oil (SO) diet
Casein	200	200
L-Cystine	3	3
Corn starch	72.8	72.8
Maltodextrin 10	100	100
Sucrose	172.8	172.8
Cellulose	50	50
Lard	179.5	0
Safflower Oil, USP	23	202.5
Mineral Mix S10026	10	10
Dicalcium phosphate	13	13
Potassium citrate, 1 H20	16.5	16.5
Choline bitartrate	2	2
Protein	24	24
Carbohydrate	41	41
Fat	24	24
Kilocalories	4057	4057
Total grams	858.15	858.15

Diet composition of Lard (LD) and Safflower Oil (SO) diets, which are 24% fat by weight (g) and 45% by kilocalories.

Table S2. Fatty acid profile of LD and SO diets.

Fatty Acid (%)	Lard (LD) diet	Safflower oil (SO) diet
Myristic acid (C14:0)	1.27 ± 0.03	0.204 ± 0.02
Palmitic acid (C16:0)	21.7 ± 0.06	7.29 ± 0.04
Palmitoleic acid (C16:1n7)	1.63 ± 0.01	0.18 ± 0.03
Stearic acid (C18:0)	11.3 ± 0.08	2.66 ± 0.24
Oleic acid (C16:1n9)	35.9 ± 0.09	14.5 ± 0.56
Linoleic acid (C18:2n6)	26.2 ± 0.17	74.8 ± 1.25
Linolenic acid (C18:3n3)	0.93 ± 0.02	0.17 ± 0.02
Eicosenoic Acid (C20:1n9)	0.55 ± 0.02	0.17 ± 0.01
Eicosadienoic acid (C20:2n6)	0.04 ± 0.00	0.04 ± 0.02

LD: Lard; SO: safflower oil; Dietary oils (n = 3 samples / group) were analyzed by gas chromatography and expressed as the mean percent ± SD of total fatty acids. All samples have a coefficient of variation of <3% between 3 experimental replicates.

Table S3. Tissue weight expressed as a percentage of total body weight and food consumption characteristics of mice after long-term feeding of LD and SO diets. .

Tissue or measurement	Lard (LD) diet	Safflower oil (SO) diet	*p*-value
Heart (% body weight)	0.393 ± 0.018	0.410 ± 0.019	0.54
Liver (% body weight)	3.82 ± 0.138	4.05 ± 0.213	0.50
Quadriceps (% body weight)	1.06 ± 0.044	1.08 ± 0.052	0.72
Gastrocnemius plantaris (% body weight)	0.790 ± 0.045	0.827 ± 0.046	0.58
Inguinal adipose (% body weight)	3.23 ± 0.456	2.42 ± 0.236	0.13

Epididymal adipose (% body weight	5.19 ± 0.339	5.55 ± 0.334	0.46
Brown adipose (% body weight)	0.511 ±.064	0.498 ± 0.057	0.88
Cumulative food intake (g)	269 ± 2.54	258 ± 6.56	0.15
Food efficiency ratio	0.014 ± 0.004	0.035 ± 0.004	0.28

LD: Lard; SO: safflower oil; data are presented as mean precent ± SEM of body weight. Food efficiency ratio is the increase in body weight (g) standardized to cumulative food intake (g). A Student's t-test was used to determine statistical significance between diet groups, with $p < 0.05$. N = 7-12/group.

Table S4. Predominant CL species in murine heart mitochondria during long-term consumption of LD or SO diets.

CL m/z ratio	Predominant acyl species	LD Diet (% of total CL)	SO Diet (% of total CL)	*p*-value
1448	(18:2)$_4$	32.8 ± 1.08	57.6 ± 0.92 *	<0.01
1450	(18:1) (18:2)$_3$	18.0 ± 0.17	18.46 ± 0.53 *	<0.01
1474	(18:1) (18:2)$_2$ (20:4)	9.51 ± 0.30	7.55 ± 0.67 *	<0.01
1498	(18:1) (18:2)$_2$ (22:6)	8.79 ± 0.30	4.55 ± 0.67 *	<0.01
1496	(18:2)$_3$ (22:6)	9.91 ± 0.51	2.50 ± 0.76 *	< 0.01
1452	(18:1)$_2$ (18:2)$_2$	7.36 ± 0.35	4.20 ± 0.18 *	0.01
1470	(18:2)$_3$ (20:5)	3.41 ± 0.05	4.41 ± 0.23 *	<0.01
1500	(18:1)$_2$ (18:2) (22:6)	3.46 ± 0.24	1.94 ± 0.26 *	<0.01
1472	(18:2)$_3$ (20:4)	3.04 ± 0.15	2.09 ± 0.11 *	<0.01
1422	(16:1) (18:2)$_3$	1.56 ± 0.10	0.75 ± 0.09 *	<0.01

LD: Lard; SO: safflower oil; data are presented as average ± SEM; an unpaired 2-tailed t-test was used to determine significant differences ($p < 0.05$) in CL species between diet groups. Asterisks indicate a significant change in CL species between diet groups; respective p-values are listed. N = 4 per group.

Table S5. Predominant CL species in murine gastrocnemius plantaris mitochondria during long-term consumption of LD or SO diets.

CL m/z ratio	Predominant acyl species	LD Diet (% of total CL)	SO Diet (% of total CL)	*p*-value
1448	(18:2)$_4$	20.17 ± 1.05	27.91 ± 1.27 *	<0.01
1450	(18:1) (18:2)$_3$	13.26 ± 0.42	14.19 ± 0.70	0.32
1452	(18:1)$_2$ (18:2)$_2$	7.98 ± 0.67	6.76 ± 0.42	0.20
1498	(18:1) (18:2)$_2$ (22:6)	6.12 ± 0.04	5.76 ± 0.31	0.32
1474	(18:1) (18:2)$_2$ (20:4)	5.95 ± 0.41	4.51 ± 0.18 *	0.03
1496	(18:2)$_3$ (22:6)	6.52 ± 0.30	4.66 ± 0.27 *	0.01
1472	(18:2)$_3$ (20:4)	5.17 ± 0.90	4.40 ± 0.37	0.48
1470	(18:2)$_3$ (20:5)	4.01 ± 1.31	5.51 ± 0.22	0.32
1476	(18:1)$_2$ (18:2) (20:4)	4.20 ± 0.23	5.15 ± 0.02 *	0.01
1500	(18:1)$_2$ (18:2) (22:6)	3.56 ± 0.07	3.12 ± 0.13 *	0.04
1426	(16:1) (18:1)$_2$ (18:2)	3.80 ± 0.16	2.78 ± 0.12 *	<0.01
1478	(18:1)$_3$ (20:4)	3.31 ± 0.12	3.26 ± 0.45	0.98
1422	(16:1) (18:2)$_3$	3.31 ± 0.45	2.86 ± 0.09	0.38
1424	(16:1) (18:1) (18:2)	3.16 ± 0.21	2.65 ± 0.04	0.08

LD: Lard; SO: safflower oil; Data are presented as average ± SEM; an unpaired 2-tailed t-test was used to determine significant differences ($p < 0.05$) in CL species between diet groups. Asterisks indicate a significant change in CL specie between diet groups; respective P-values are listed. N = 3 per group.

Table S6. Predominant CL species in murine inguinal white adipose tissue (iWAT) during long-term consumption of LD or SO diets.

CL m/z ratio	Predominant acyl species	LD Diet (% of total CL)	SO Diet (% of total CL)	*p*-value
1448	$(18:2)_4$	35.03 ± 1.19	48.87 ± 1.65 *	<0.01
1450	(18:1) $(18:2)_3$	18.98 ± 0.63	15.57 ± 0.60 *	<0.01
1452	$(18:1)_2$ $(18:2)_2$	10.51 ± 0.33	6.73 ± 0.19 *	<0.01
1422	(16:1) $(18:2)_3$	6.55 ± 0.41	6.41 ± 0.33	0.61
1474	(18:1) $(18:2)_2$ (20:4)	4.58 ± 0.74	4.13 ± 0.29	0.59
1470	$(18:2)_3$ (20:5)	3.92 ± 0.18	4.48 ± 0.48	0.32
1472	$(18:2)_3$ (20:4)	3.92 ± 0.35	3.32 ± 0.07	0.14
1424	(16:1) (18:1) (18:2)	3.87 ± 0.34	2.51 ± 0.18	0.01
1496	$(18:2)_3$ (22:6)	3.10 ± 0.75	2.29 ± 0.78	0.48
1500	$(18:1)_2$ (18:2) (22:6)	2.47 ± 0.57	1.98 ± 0.28	0.46
1454	$(18:1)_3$(18:2)	3.11 ± 0.39	1.15 ± 0.10 *	<0.03
1456	$(18:1)_4$	2.64 ± 0.30	1.57 ± 0.36	0.059
1498	(18:1) $(18:2)_2$ (22:6)	1.32 ± 0.27	1.11 ± 0.18	0.54

LD: Lard; SO: safflower oil; Data are presented as average ± SEM; an unpaired 2-tailed t-test was used to determine significant differences ($p < 0.05$) in CL species between diet groups. Asterisks indicate a significant change in CL species between diet groups; respective p-values are listed. N = 4 per group.

Table S7. Predominant CL species in murine epididymal white adipose tissue (eWAT) during long-term consumption of LD or SO diets.

CL m/z ratio	Predominant acyl species	LD Diet (% of total CL)	SO Diet (% of total CL)	*p*-value
1448	$(18:2)_4$	29.50 ± 1.91	32.55 ± 1.23	0.65
1450	(18:1) $(18:2)_3$	17.39± 0.48	14.72 ± 1.23	0.09
1452	$(18:1)_2$ $(18:2)_2$	9.52 ± 1.28	68.74 ± 0.72*	0.62
1474	(18:1) $(18:2)_2$ (20:4)	5.50 ± 0.31	6.32 ± 0.88	0.39
1456	$(18:1)_4$	5.33 ± 1.38	5.72 ± 1.48	0.85
1422	(16:1) $(18:2)_3$	6.58 ± 1.01	4.11 ± 0.31	0.06
1496	$(18:2)_3$ (22:6)	5.17 ± 2.76	4.54 ± 1.69	0.39
1500	$(18:1)_2$ (18:2) (22:6)	4.17 ± 0.79	5.37 ± 1.53	0.51
1472	$(18:2)_3$ (20:4)	4.37 ± 0.44	4.80 ± 0.67	0.61
1454	$(18:1)_3$(18:2)	4.78 ± 0.65	3.84 ± 1.04	0.85
1470	$(18:2)_3$ (20:5)	3.45 ± 0.46	3.82 ± 0.26	0.51
1424	(16:1) (18:1) (18:2)	2.49 ± 0.46	2.54 ± 0.14	0.093

LD: Lard; SO: safflower oil; Data are presented as average ± SEM; an unpaired 2-tailed t-test was used to determine significant differences ($p < 0.05$) in CL species between diet groups. Asterisks indicate a significant change in CL species between diet groups; respective p-values are listed. N = 4 per group.

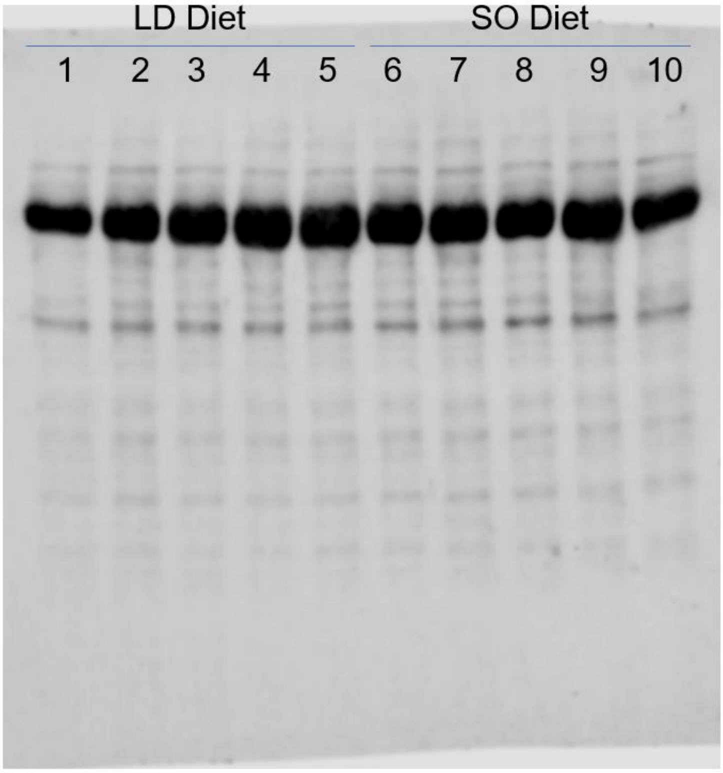

Lane	Total protein intensity
1	28.1
2	33.6
3	32.5
4	33.6
5	34.0
6	30.7
7	35.3
8	33.9
9	38.1
10	32.4

Figure S1. Total protein stain used as a loading control for OXPHOS western blot on BAT mitochondrial extracts. Protein intensity was measured using Revert 700 total protein stain (Licor Biosciences, Lincoln, NE, USA), detected using the Licor Odyssey Imager, and densitometric analysis was carried out using the Licor Imager Software (Lincoln, NE, USA). OXPHOS complex protein intensity was standardized to the total protein intensity for each well for relative protein abundance quantified in Figure 4b.

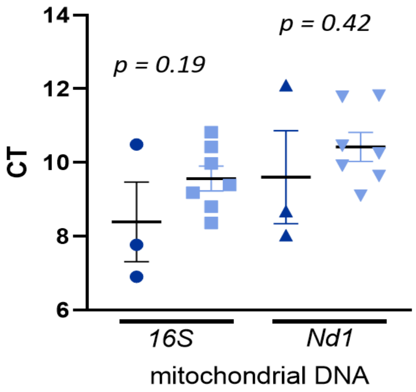

Figure S2. Mitochondrial DNA quantification of *mtNd1* and *mt16S* from representative technical replicates to validate relative amounts of mitochondria between samples. Data are expressed as mean ± SEM. Two-sample t-tests were used to determine significant differences between diet groups, with $p < 0.05$. *p*-values are listed in the figure.
